# Differential Effects of Heated Perfusate on Morphology, Viability, and Dissemination of Staphylococcus epidermidis Biofilms

**DOI:** 10.1128/AEM.01193-20

**Published:** 2020-10-01

**Authors:** Joanne K. Beckwith, J. Scott VanEpps, Michael J. Solomon

**Affiliations:** a Department of Chemical Engineering, University of Michigan, Ann Arbor, Michigan, USA; b Department of Emergency Medicine, University of Michigan, Ann Arbor, Michigan, USA; c Biointerfaces Institute, University of Michigan, Ann Arbor, Michigan, USA; d Department of Biomedical Engineering, University of Michigan, Ann Arbor, Michigan, USA; e Macromolecular Science and Engineering, University of Michigan, Ann Arbor, Michigan, USA; f Michigan Center for Integrative Research in Critical Care, University of Michigan, Ann Arbor, Michigan, USA; University of Manchester

**Keywords:** *Staphylococcus epidermidis*, biofilm structure, biofilm detachment, biofilms

## Abstract

The biofilm phenotype offers bacterial communities protection from environmental factors, as evidenced by its role in the viability, persistence, and virulence of cells under conditions in which flow is present, such as in riverbeds, industrial piping networks, and the human circulatory system. Here, we examined the hypothesis that temperature—an environmental factor that affects the growth of the Gram-positive bacterium Staphylococcus epidermidis—controls, through dual mechanisms, persistence of this bacterial strain in a shear environment characteristic of the human circulatory system. We demonstrated that temperature and antibiotics impact the surface-adhered biofilm and material disseminated downstream in different ways. Specifically, by means of three-dimensional (3D) confocal and scanning electron microscopy, an increase in surface-adhered biofilm heterogeneity was observed with increasing temperature. Additionally, we found a 4-log decrease in cell viability at the biofilm surface as the perfusate temperature was increased from 37°C to 50°C. Finally, the viability of cell-containing fragments that were disseminated from the substrate was assessed by downstream sampling, culture, and optical density measurement. We found that although temperature decreased the viability of the surface-adhered biofilm, the downstream material remained viable. And yet, in the presence of antibiotics, the growth of disseminated material was nearly completely inhibited, even though the addition of antibiotics had no significant impact on the viability of the surface-adhered biofilm. The mechanism involves both biofilm structural damage, as quantified by morphology of debrided material, and reduced cell viability, as quantified by assay of bacterial cells present in the surface-adherent biofilm and in the downstream effluent. The results potentially identify parameter ranges in which elevated temperature could augment current antibiotic treatment regimens.

**IMPORTANCE** Bacterial biofilms are a leading cause of medical device infections. Staphylococcus epidermidis is commonly responsible for these types of infections. With increasing occurrences of antibacterial resistance, there has been a new push to explore treatment options that augment traditional antibiotic therapies. Here, we show how thermal treatment can be applied to both degrade bacterial biofilms on substrates and impede the proliferation of cells that detach from them. Understanding the response of both surface-adhered and dispersed bacterial cells under thermal stress conditions is a foundational step toward the development of an *in situ* treatment/remediation method for biofilm growth in medical devices; such an application could use oscillatory flow of heated fluid in a catheter as an adjuvant to antibiotic treatment. The work furthermore provides new insight into the viability of disseminated biofilm material.

## Abstract (precis)

Bacterial biofilms are a leading cause of medical device infections. Staphylococcus epidermidis is commonly responsible for these types of infections. With increasing occurrences of antibacterial resistance, there has been a new push to explore treatment options that augment traditional antibiotic therapies. Here, we show how thermal treatment can be applied to both degrade bacterial biofilms on substrates and impede the proliferation of cells that detach from them. Understanding the response of both surface-adhered and dispersed bacterial cells under thermal stress conditions is a foundational step toward the development of an *in situ* treatment/remediation method for biofilm growth in medical devices; such an application could use oscillatory flow of heated fluid in a catheter as an adjuvant to antibiotic treatment. The work furthermore provides new insight into the viability of disseminated biofilm material.

## INTRODUCTION

Biofilms are microbial communities that often develop at solid-fluid interfaces. The bacterial cells exist in a sessile phenotype that is adherent to a solid surface (1). Biofilms are adaptive (2) and able to survive harsh conditions as well as antibiotic treatments that would otherwise eradicate planktonic cells (3). Among the factors contributing to this resilience are the extracellular polymeric substances (EPS)—polysaccharides, proteins, nucleic acids, lipids, and other biopolymers—that form a matrix in which the bacterial cells are suspended (4). Three hypotheses explaining the refractory nature of biofilms are as follows: (i) the decreased diffusion and increased deactivation of antibiotics through interaction with the EPS; (ii) the formation of oxygen- and nutrient-depleted microenvironments, which reduce metabolic activity, decreasing antibiotic uptake and/or efficacy; and (iii) the maintenance of a subpopulation of cells in a highly protected, quiescent phenotypic state (i.e., persister cells) (5).

Notwithstanding this resilience, recent work has indicated that biological and physical properties of biofilms respond to temperature in unexpected ways. For example, it has been previously demonstrated that staphylococci are killed at elevated temperatures of 40°C and 45°C (6); hyperthermia also augments the effectiveness of antibiotics in both planktonic and biofilm-embedded bacteria (6, 7). In addition, the elastic modulus of biofilms is temperature sensitive; staphylococcal biofilms experience irreversible softening above 45°C (8). Finally, the yield stress of biofilms is sensitive to temperature; the structural integrity of the biofilm is weakened significantly by elevations in temperature (9). For example, the yield stress of a staphylococcal biofilm decreased to 3.9 Pa at 60°C compared to 23.3 Pa at 37°C.

In addition to temperature, shear stress generated by fluid flow has been suggested to impact biofilm morphology, attachment, and dispersal. For example, a study combining simulation and experiment showed that biofilms grown under static conditions formed mushroom-type structures, while those grown under conditions of high shear rate flow formed elongated streamers (10). Shear stress also plays a role in attachment of Pseudomonas aeruginosa biofilms; when grown under high shear rate flow conditions, they are more cohesive and strongly attached to the substrate than those grown under low shear rate flow conditions (11). It is also believed that shear stress may impact the detachment of bacterial cells within the biofilm. The two mechanisms of cellular detachment have been described as sloughing—in which the organism’s phenotype drives shedding of cells—and erosion—in which cellular material is mechanically debrided from the substrate (12).

There have been a number of attempts to apply heat to biofilms in a clinical setting, including the use of magnetic nanoparticles (13), radiofrequency ablation (14), and laser irradiation (15). In our work, we explored the use of a heated perfusate to deliver heat (16). An approach to heat treatment based on flow of perfusate at elevated temperatures could have applications in the treatment of hemodialysis catheter infections.

These research results, which addressed the effects of temperature and shear stress in isolation, can be the basis of further study, particularly with respect to correlative effects of elevated temperature and shear stress on cell viability and biofilm morphology and their corelationship on bacterial persistence in flowing systems. Here, “persistence” refers to the viability of surface-adhered bacteria and the ability of disseminated bacteria to repopulate, following a treatment regimen. Specifically, prior work has implicated the effect of shear flow in biofilm characteristics such as attachment and dispersion; this parameter should therefore be controlled in any study designed to understand the role of temperature in biofilm morphology and the downstream dissemination of biomass from it. Additional scientific issues can be addressed by assessing the degree of temperature-induced disruption of biofilm morphology, as well as the degree of cell death at the site of biofilm attachment. Finally, the amount of temperature-induced debridement, and the ultimate viability of cells that have been debrided, should be assessed.

The purpose of this work is therefore to evaluate the hypothesis that elevated temperature affects bacterial persistence through dual mechanisms that involve both damage to biofilm morphology and reduction in cell viability and that the susceptibility to elevated temperature of biofilm material attached to a substrate is different from the susceptibility of material disseminated during heat treatment. This issue is examined in a staphylococcal biofilm grown under well-controlled shear (i.e., flow) and temperature conditions. The specific questions addressed by the study are as follows. First, what are the effects of elevated temperature on the morphology and cell viability of a surface-adherent biofilm under flowing conditions? Second, when exposed to elevated temperature owing to proximate flow of a heated fluid, what is the viability of the cells that become detached from the biofilm? Finally, for both the surface-adhered biofilm and detached cells, are the effects of elevated temperature on morphology and viability impacted by the additional presence of antibiotics?

Biofilms present on implanted medical devices, such as hemodialysis catheters (17), represent an important instance where shear stresses and elevated temperatures could play a role in cellular growth and dispersal. Shear stress and perfusate flow in such devices replicate conditions of biofilm flow cell experiments in literature (16). This study sought to understand the response of bacterial biofilms exposed to elevated temperature in a flow cell model of clinical relevance. As previously described, cells enmeshed in biofilms can have reduced metabolic activity; this reduced activity can contribute to reduced antibiotic effect (18, 19). Likewise, biofilm development (particularly by Staphylococcus epidermidis) may go largely unchecked by the host immune system, owing to multiple immune evasion properties (20, 21). However, during the detachment stage of the biofilm life cycle, planktonic cells (22) are shed from the surface into the bloodstream, potentially causing bloodstream infections or metastatic spread elsewhere in the patient (2). With no current means available to eradicate the infection *in situ*, current treatment recommendations include the removal and replacement of the infected device (23–25), which entails significant morbidity and mortality (26). Moreover, the average cost of treatment of one catheter-associated infection in the United States in 2012 was $70,696 (27). This report therefore provides a scientific foundation for the exploration of treatment methods incorporating the physical variables of temperature/heat and fluid shear in addition to the appropriate use of antibiotics. This variable has long been used *ex vivo* to control microbial growth in both medical and nonmedical settings through methods such as autoclaving and pasteurization.

## RESULTS

### Qualitative observations.

**FIG 1:**
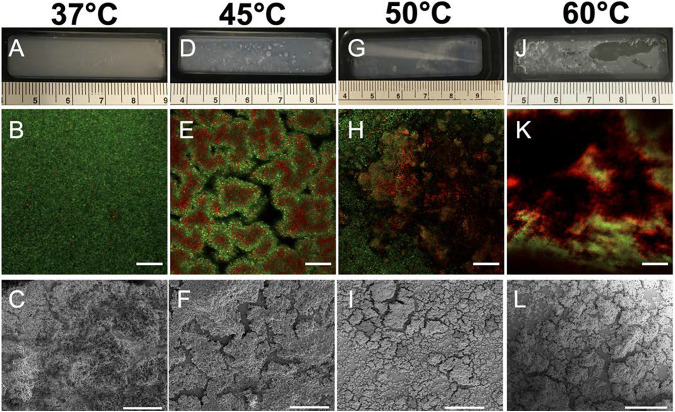
Effect of temperature retreatment on biofilm morphology at multiple scales. (A to C) Flow cell, CLSM, and SEM images of cell morphology were acquired for biofilm exposed to 37°C fluid flow as shown in panels A, B, and C, respectively. (D to L) These images were also collected for biofilms exposed to 45°C (D to F), 50°C (G to I), and 60°C (J to L) perfusate. Ruler markings in panels A, D, G, and J are in centimeters. Scale bars are 50 μm for confocal images (panels B, E, H, and K) and 30 μm for SEM images (panels C, F, I, and L).

As shown in Fig. 1A to C, biofilms exposed to the 37°C condition presented as a thick, confluent layer. The distribution of cells was approximately uniform in the plane parallel to the shear surface as well as along an axis normal to it. Macroscopically, the biofilm surface appears smooth with no visible deformity (Fig. 1A). Confocal microscopy shows that the majority of the cells at 37°C still had intact cell membranes as evident by the small number of cells stained by propidium iodine (Fig. 1B). Scanning electron microscopy (SEM) results confirmed that the biofilm surface had a homogenous distribution of cells (Fig. 1C).

When the temperature was elevated to 45°C, we observed pilling on the surface of the biofilm and the formation of streamers (Fig. 1D). Some microheterogeneity (clustering and aggregation) of cells is apparent in the high-resolution confocal laser scanning microscopy (CLSM) image volumes. These deformities are themselves heterogeneous on the microscopic scale (Fig. 1E and F); CLSM imaging indicates the presence of mound structures with void space between the mounds. These voids presumably arise because cells had detached from the surface in these regions. Interestingly, these mound structures appear to have patterning of live and dead cells, with the dead cells concentrated on the interior of the mound, while the outer mound surface appears to have been alive as evident in confocal images in Fig. 1E. The outer ring of living cells raises interesting issues about the viability of the cells that had detached to produce the voids.

As the temperature was increased to 50°C, the visible deformities became more extreme, as made evident by the detachment of biofilm from the substrate surface at the entrance of the flow cell (Fig. 1G). On a microscopic scale, we observed that the mound structures had begun to break up, leaving large areas of the substrate with few cells attached (Fig. 1H and I).

At 60°C, we saw a complete disruption of the biofilm. Visually, large portions of the biofilm had been shed from the surface (Fig. 1J). Microscopic imaging indicates that there were no cells adhered in these regions (Fig. 1K and L). Large portions of the cells that remain adhered to the substrate surface at 60°C (and at 50°C) appeared to have compromised cell membranes, as evidenced by the red regions in Fig. 1H and K. This suggests a high degree of cell death in biofilms subjected to these temperatures. The CLSM observations of structure are corroborated by scanning electron microscopy. Specifically, cells begin to form mounds and eventually become detached from the substrate surface when exposed to elevated temperatures, as evident in Fig. 1I and L.

### Cell viability.

**FIG 2:**
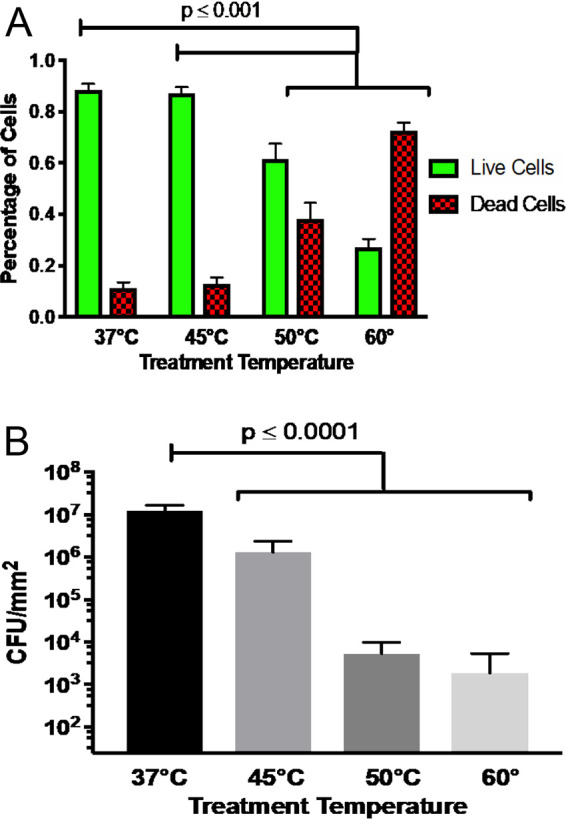
Cell death and viability in biofilm after temperature treatment. (A) Quantified image analysis showing the proportions of live and dead cells after a 2-h treatment at 37°C, 45°C, 50°C, and 60°C. Green indicates live cells, while red indicates dead cells. All comparisons yielded a *P* value of <0.001, except for the difference between 37°C and 45°C, which was not statistically significant. (B) Quantitative culture of cellular material collected from the entire surface-adhered biofilm. The graph origin represents the minimum level of detection, 24 CFU/mm^2^. *P* values represent <0.0001 for results determined at 45°C, 50°C, and 60°C compared to baseline 37°C. All error bars are standard errors of the means.

Observations of the number of cells stained red and green, which can be correlated with cell viability of the surface-adherent microbial community, were quantified (Fig. 2A). The average number of cells in a 30-by-30-by-10-μm image volume was counted to obtain a number density of live (green) and dead (red) cells. The average total number of cells in each sample was approximately 1,000. At 37°C, 89% of the cells in an image volume were green, while 11% were red. At 45°C, there was a slight decrease in cell viability, with 87% of the cells appearing green and 13% staining red. The decrease in cell viability was more evident at 50°C, where 64% of the cells stained green with 36% red, and at 60°C, where 27% of the cells were green and 73% red. These results support the hypothesis that as temperature increases there is an overall decrease in cell viability. The viable cell densities at the four temperatures, 37°C, 45°C, 50°C, and 60°C, were 0.11 ± 0.02, 0.062 ± 0.007, 0.044 ± 0.007, and 0.036± 0.006 cells per μm^3^, respectively. Levels of cell viability for the 50°C and 60°C treatments were significantly different from those seen for the control and 45°C treatment (*P* < 0.001).

These trends in cell viability resulting from live/dead staining were confirmed using quantitative culture (Fig. 2B). At 37°C, the biofilm produced 1.2 × 10^9^ CFU per mm^2^. There was a 1-log reduction for biofilms treated at 45°C (1.3 × 10^8^ CFU per mm^2^). Biofilms treated at 50°C and 60°C had nearly 4-log reductions (3.5 × 10^5^ CFU per mm^2^ and 1.8 × 10^5^ CFU per mm^2^, respectively) compared to 37°C. Statistical significance was evaluated using one-way analysis of variance (ANOVA) with multiple comparisons to baseline 37°C results. The results determined at all three treatment temperatures were significantly different from the 37°C results (*P* < 0.0001). Although the quantitative culture results confirm the general trend of decreased cell viability at elevated temperatures per live/dead staining, the numerical values of live and dead cells were not identical for the two methods. The difference could be due to three potential factors. First, because the CLSM cell counting is performed in a known volume, a number density per unit volume can be computed, while the quantitative culture is provided as a number density per unit area with the thickness of the biofilm unknown. Second, live/dead staining and quantitative culture are different measures, with the former providing an indirect assessment of viability based on membrane integrity and the latter providing a direct measure of the cells’ ability to be cultured. Third, edge effects on flow and heat transfer may affect the thermal treatment in these regions of the flow cell. The fact that confocal microscopy imaging avoids these regions and quantitative culture includes them could lead to variability in the two measurements.

### Structural analysis.

**FIG 3:**
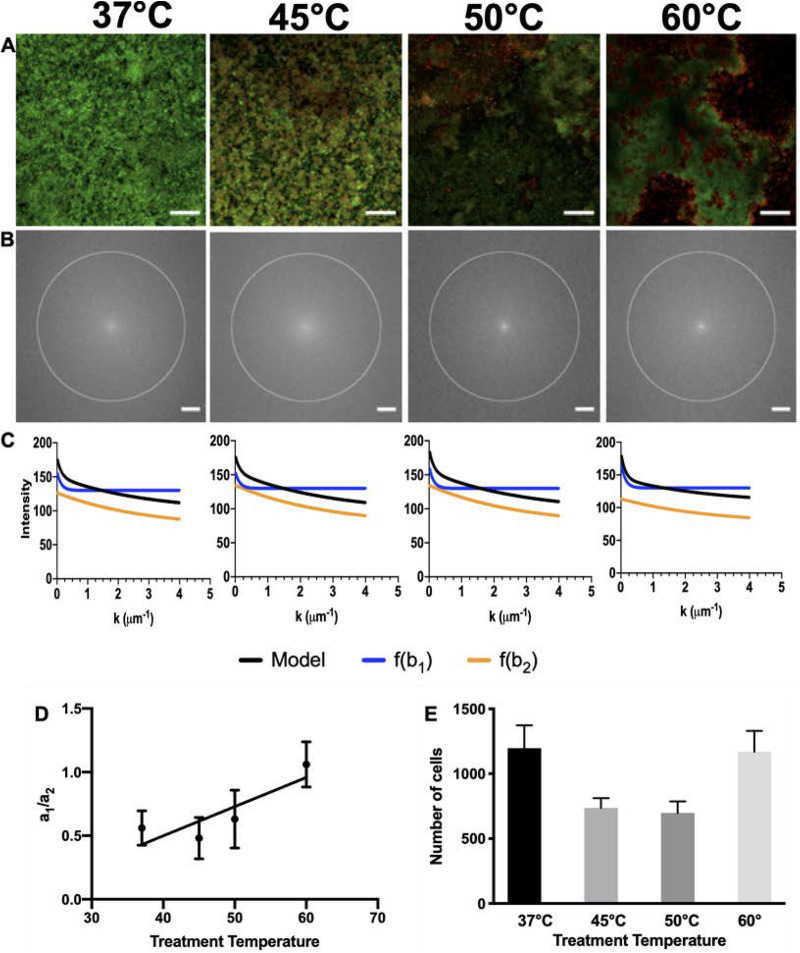
Large-scale morphology of biofilm after temperature treatment. (A and B) Typical CLSM images (A) and fast Fourier transform (FFT) results (B). The scale bars for panels A and B are 50 μm and 50 pixels, respectively. A circle with a radius of 200 px has been added to panel B, representing the largest radial distance at which the FFT intensity values were averaged. The panel B results were used to generate panel C, which shows the dependence of the FFT on the spatial frequency, *k*. The black curve represents the full-model equation fit, while the blue curve represents the contribution from the larger-length scale and the orange curve represents the smaller, cellular-length scale. In panel C, the data represent averages of results from three replicates. (D) The ratios of the amplitude coefficients for the large- and small-length-scale structures, *a*_1_ and *a*_2_, respectively, are plotted. Error bars are standard errors of the means. The linear regression has a *P* value of 0.056. Coefficients *a*_1_ and *a*_2_ are defined in equation 1. (E) The average number of cells (both live and dead) under each temperature condition, as resolved in a 30-by-30-by-10-μm image volume.

The microheterogeneity qualitatively observed through CLSM and SEM was quantified using fast Fourier transform (FFT) analysis. By converting large-scale confocal micrographs to the spatial frequency domain (Fig. 3A and B), the pixel intensity as a function of the spatial frequency, *k*, was plotted (Fig. 3C). The isotropy of the FFT warrants use of a scalar *k*, where *k* represents an inverse length scale. FFT intensity at low *k* corresponds to structure on long spatial scales; high *k* reflects spatial structure on small scales. The FFT data at each test temperature was first fitted to a five-parameter, double-exponential model:

(1) $$f\left(k\right)=a_{1}e^{-b_{1}k}+a_{2}e^{-b_{2}k}+d$$

In this model, *b*_1_ and *b*_2_ represent length scales and *a*_1_ and *a*_2_ represent the weighted contributions of each length scale. Following the first fit, the average values of *b*_1_ and *b*_2_ were calculated as 7.6 μm and 0.35 μm, respectively. The model was then recalculated, fixing *b*_1_ and *b*_2_ at these values for all specimens, so as to parsimoniously determine the characteristic ratio of amplitudes *a*_1_/*a*_2_. As shown in Fig. 3D, there was a trend such that as temperature increased, this ratio of coefficients for the large (∼7-μm) and small (∼0.3-μm) length scales, *a*_1_ and *a*_2_, respectively, also increased. Physically, the increasing trend in *a*_1_/*a*_2_ with temperature is indicative of an increase in heterogeneity, with a larger contribution of pixel intensity coming from the large length scale. However, this trend does not quite reach statistical significance (*P* = 0.056 for the hypothesis that the slope of the regression line differs from zero). The full set of fit parameters *a*_1_, *a*_2_, *b*_1_, *b*_2_, and *d* for both the initial fit and the fit with fixed *b*_1_ and *b*_2_ can be found in Tables SI.2 and SI.3 in the supplemental material, respectively.

Figure 3E demonstrates the number density of cells, i.e., the average number of cells (both live and dead) under each temperature condition, as resolved in a 30-by-30-by-10-μm image volume. This is an additional measure of biofilm morphological change. The number density of cells decreased at moderate temperatures (45°C and 50°C) and then recovered at the highest temperature of 60°C. The difference between the number of cells at 37°C and 60°C was minimal.

### Downstream effects.

**FIG 4:**
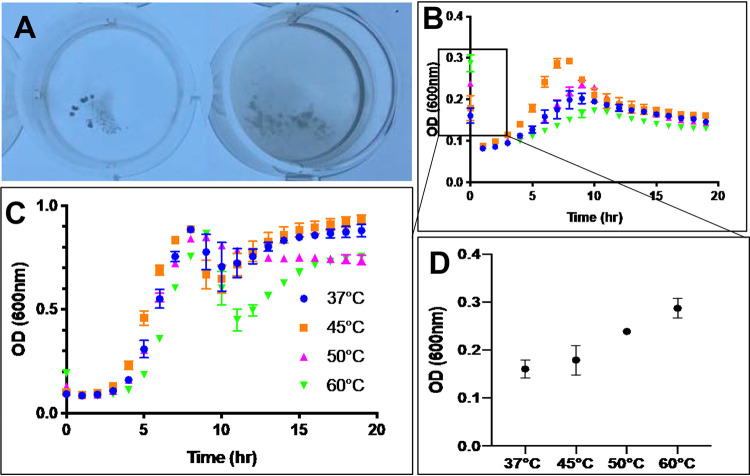
Viability of cells collected from the flow cell effluent stream after temperature treatment. (A) Representative image of bacterial growth in 2 ml effluent (left) and 1 ml effluent plus 1 ml fresh TSBG media (right) after 18 h. The well diameter was 2.6 cm. Growth curves determined under these conditions are shown in panels B and C, respectively. The initial OD_600_ was measured from the curve shown in panel B and in plotted in panel D. All error bars are standard errors of the means.

The viability of sloughed or debrided biomass was evaluated by growth curve analysis of effluent from the flow cell that had been sampled downstream. Figure 4A shows a photograph of the microwell plate after the 18-h incubation of this effluent at 37^0^C. Bacteria with no added media (left) appeared to aggregate, with the effluent media solution surrounding them remaining clear. When fresh medium was added to the effluent (right), the bacterial aggregates still formed; however, the media also became cloudy, suggesting that both planktonic cells (cloudy media) and biofilm material (bacterial aggregates) were present. The resulting growth curves are presented in Fig. 4B and C for the effluent-only culture and for the effluent with fresh media, respectively. Values representing the initial optical density at 600 nm (OD_600_), as taken from Fig. 4B, are plotted in Fig. 4D. The data show that there was an increase in the amount of initial biomass as the temperature increased. Despite this increase in initial biomass, the growth curves in Fig. 4C show there was no significant change in the rate of cellular division or the maximum OD_600_ with increasing temperature.

### Addition of antibiotics.

**FIG 5:**
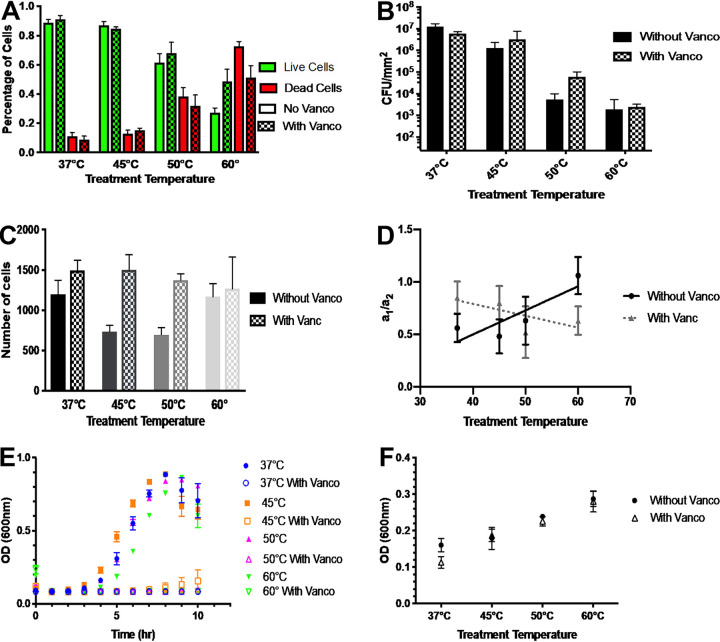
Additional effect of vancomycin on biofilm and effluent viability and morphology. Each graph contains two sets of data. The first set was determined in the presence of the antibiotic in addition to heat treatment (indicated by “With Vanco” in each figure legend). The second set represents the results seen after heat treatment alone and has been reproduced for comparison from the data presented in Fig. 2A, 2B, 3E, 3D, 4C, and 4D for panels A, B, C, D, E, and F, respectively. (A) Quantified image analysis showing the proportions of live and dead cells after a 2-h treatment at 37°C, 45°C, 50°C, and 60°C. Green indicates live cells, while red indicates dead cells. (B) Quantitative culture of cellular material collected from the entire surface-adhered biofilm. The graph origin represents the minimum level of detection, 24 CFU/mm^2^. (C) The average number of cells (both live and dead) under each temperature condition, as resolved in a 30-by-30-by-10-μm image volume. (D) The ratios of the amplitude coefficients for the large- and small-length-scale structures, *a*_1_ and *a*_2_, respectively, are plotted. (E) Ten-hour growth curves for 1 ml effluent with 1 ml fresh TSBG media. (F) The initial OD_600_ was measured from the curve representing 2 ml effluent (Fig. SI.2). All error bars are standard errors of the means.

Figure 5 depicts a case study where the heat treatment fluid was supplemented with 30 mg/ml of the antibiotic vancomycin. Panels A and B of Fig. 5 show the impact of heat and vancomycin on the viability of the surface-adhered biofilm. For this biofilm, there was no additional killing benefit seen from the addition of vancomycin. This finding is equally well indicated by live/dead staining (Fig. 5A) and by quantitative growth culture (Fig. 5B). Panels C and D of Fig. 5 show that the addition of antibiotic may have minimized the effect of heat on the structure of the biofilm, because its introduction generated no change in the total number of cells in the system (Fig. 5C) as well as no resolvable change in heterogeneity, as reported by the FFT characterization (Fig. 5D).

Antibiotics did, however, have a significant impact on the viability of cells collected in the downstream effluent. As shown in Fig. 5E, the 37°C, 50°C, and 60°C effluent had zero growth of the disseminated bacteria when added to 1 ml of fresh tryptic soy broth (TSB) medium supplemented with 1% d-(+)-glucose (TSBG). The effluent maintained at 45°C showed slight growth; however, this growth occurred at a much lower rate than was seen with the effluent treated with heat alone. Additionally, vancomycin had no impact on the initial OD_600_ of the downstream effluent. Specifically, the initial amount of cellular material in the antibiotic-treated effluent was the same as for the effluent treated with heat alone.

## DISCUSSION

This paper has shown the effect of elevated temperature on the viability and the morphology of S. epidermidis biofilms and their disseminated biomasses. Our study was designed to explore the impact of a heated fluid on the morphology and viability of surface-adhered biofilms and the biomass disseminated from the surface. We demonstrated that there are differential impacts of temperature on these two cell populations. The viability of the surface-adhered biofilm is susceptible to heat; however, the cellular material that is released from the biofilm into the effluent stream is viable. Antibiotics introduce additional impacts. Although antibiotics have little impact on the surface-adhered biofilm at any temperature, they significantly reduce the viability of the detached material. In this section, we first address the utility of the fast Fourier transform (FFT) image analysis used in this study for morphological quantification. Next, we discuss temperature effects in light of the different mechanisms of biofilm detachment. Then, we explore the synergistic effects of elevated temperature and antibiotic concentration. We conclude by describing the directions for future studies and the significance of our findings for potential treatments.

In order to quantify the heterogeneity observed in confocal microscopy, we performed FFT analysis of microscope images of biofilm in the flow cell. This technique can enjoy application beyond the present study because the method is simple and can be easily automated. This method allows direct quantification of changes in biofilm structure observed qualitatively in microscopy images. This technique could potentially be applied to, for example, live-cell imaging of the time-dependent formation of biofilms.

The FFT method quantified morphological change that was indicative of cellular debridement. We argue that the biofilms in our system appeared to undergo both sloughing (organism-driven detachment) and erosion (mechanically driven detachment) (12); additionally, sloughed cells are in the planktonic phenotype, while eroded cells may persist in the biofilm phenotype (22). Temperature was observed to affect which detachment mode dominated. Specifically, sloughing was dominant at low temperature and erosion at high temperatures. Moreover, the functional dependences of the mechanism on temperature differed; sloughing occurred at all temperatures, while the degree of erosion was a function of increasing temperature. The conjecture that sloughing and erosion dominate at different temperatures is supported by the optical density (OD) data. These data show that the growth rate of effluent cells was independent of the amount of material disseminated. The conjecture is further corroborated by the spatial patterning of the gaps in the confocal microscopy images of the disrupted biofilms. Finally, it is consistent with the literature on the effects of heat on biofilm yield stress. Each set of evidence is described below.

The primary set of evidence is the consistent growth rate of effluent cells regardless of the initial amount of disseminated material in the sample. This claim is supported by growth curve analysis. Figure 4D shows that the initial amount of material disseminated, both sloughed and eroded, increased with temperature. Figure 4C shows that there was little difference in the growth rates of cells exposed to elevated temperature, regardless of the OD of the starting material in the disseminated material. This suggests that the amount of culturable material shed by the biofilm was constant under all temperature conditions and that the excess material observed by optical density at elevated temperatures was actually dead, nonviable cells. Although the overall OD_600_ was higher at the beginning of the growth curve analysis, this dead material settled out of solution over time and only the live, viable material contributed to the turbidity measured in the growth curve. Temperature decreased the viability of the bacterial cells in the surface-adhered biofilm, but it had limited impact on the ability of the detached cells to repopulate in the planktonic state once disseminated and returned to room temperature.

Corroborating evidence supporting the temperature-dependent nature of detachment can be found in the heterogenous breakup of the biofilm observed under confocal microscopy as shown in Fig. 1. At 37°C and 45°C, the areas where cells had become detached from the surface (i.e., the clusters evident in Fig. 1E) were surrounded by viable cells; however, at 50°C and 60°C, many of the regions of biofilm breakup contained mostly dead cells. It is conceivable then that, at 37°C and 45°C, individual cells were released from viable regions of biofilm clusters into the effluent. As the temperature increased, the surface-adhered biofilm began to die. Therefore, any additionally biofilms that became detached were nonviable; these cells contributed to the initial OD_600_ readings but not to the growth curve.

Finally, the hypothesis that sloughing and erosion occur in tandem is supported by previous research on the yield behavior of biofilm materials. Sloughing is a bioactive process that occurs in response to environmental stress; it does not require mechanical degradation and can occur under quiescent conditions (12). Erosion, however, requires a mechanical force to disrupt the biofilm material, causing yielding. As temperature is increased, the stress required for yielding decreases (9), creating an environment where erosion can more predominately occur. These results suggest that yield stress not only impacts the structural integrity of the biofilm material but also plays a role in the ability of the biofilm to remain attached to a surface and become disseminated under flowing conditions.

We next turn to the synergistic effects of temperature and antibiotics on biofilm morphology and viability. The antibiotic case study presented in Fig. 5 shows that antibiotics had little impact on the viability of the surface-adhered biofilm (Fig. 5A and B) but had a very large impact on the viability of the material disseminated downstream (Fig. 5E), preventing almost all regrowth in this case. This is consistent with other studies that have shown that biofilms are less susceptible to antibiotics than freely floating cells (5, 18). Figure 5F, however, shows that the same amounts of material were disseminated with and without antibiotics. This further supports the hypothesis that planktonic cells are sloughed at all temperatures and are thus susceptible to antibiotics. As the treatment temperature increases, large amounts of dead biofilm material are eroded off the biofilm into the environment.

The data representing the effects that antibiotics have on biofilm morphology at high temperature are inconclusive. As shown in Fig. 5C and D, the case of added antibiotics did not display the same temperature-dependent dip in cell density or increase in heterogeneity as was observed for the case of heat treatment alone. This suggests that the effects of the dual impacts of heat and antibiotics are more complicated than would be expected based on a simple addition of the two independent effects of heat and antibiotic. Instead, there is coupling between the two. To that end, it would be of interest to investigate the effects of antibiotics across a broader range of concentrations. (The concentration of antibiotic used in the study was chosen to represent the high end of a clinical dose peak.) Such a study could consider antibiotic concentrations extending from the MIC of 2 mg/liter to the high end of clinical doses at or above the 30 mg/liter used in this study.

Future work could also investigate the gene expression and phenotypic make-up of cells disseminated under each set of temperature and antibiotic conditions. As shown in Fig. 4C, there was no difference in the growth curves of disseminated material up to 10 h. Although 10 h is a common time at which growth curves are often truncated, in the present study we allowed cells to grow for an additional 8 h, because 18-h duration is sufficiently long to be relevant to biofilm formation (28). When the cells were allowed to grow for a total of 18 h, a second growth phase appeared to initiate at around 10 h and continued until the end of the measurement. We speculate that the observation of a second growth phase may indicate that the disseminated material had the ability to reestablish itself as a biofilm. In this second phase, it was observed that the cells at higher temperatures had lower growth rates and lower maximum ODs than those exposed to lower temperatures. This trend suggests that the heated biofilms may have had a diminished biofilm-forming capacity. This speculative analysis could be investigated through future work. Such work could investigate if there is a transcriptional shift occurring at 10 h and later; such a shift would confirm the qualitative phenotypic shift about which we have hypothesized. Such work would provide valuable insight into the infection potential of these bacteria; that is, it would establish if the cells could cause planktonic or metastatic biofilm infections.

In conclusion, we have established that temperature has a dual effect on bacterial biofilms, affecting both the surface-adhered biofilm material and the cellular material disseminated downstream. These effects occur when the biofilm is exposed to continuous flow and persist when antibiotics are also administered. When exposed to heat, the biofilm undergoes both sloughing and debridement. Sloughing occurs under all exposure temperatures, while debridement is more common at elevated temperatures. Elevated temperature decreases the viability of surface-adhered biofilms but has little impact on the ability of disseminated material to regrow. There is a preliminary indication that temperature might impact the ability of disseminated material to form a biofilm. Adding antibiotics to the temperature treatment had little impact on the surface-adhered biofilm; however, at all temperatures the antibiotics were more effective at killing cells in the disseminated material than heat alone was capable of. This work provides valuable insight into the determination of the therapeutic window within which heat could be effective as an *in situ* biofilm treatment; a potential application could use oscillatory flow of heated fluid through a catheter as an adjuvant to antibiotic treatment.

## MATERIALS AND METHODS

### Bacterial strain.

Staphylococcus epidermidis was used as the model bacteria in this study. It is the most common pathogen associated with catheter infections (29). S. epidermidis is a coagulase-negative staphylococcal species that is a ubiquitous colonizer on human skin and mucosal membranes. It is an important opportunistic pathogen (21). S. epidermidis RP62A was obtained from American Type Culture Collection (ATCC 35984), stored at –80°C as a glycerol stock, streaked onto on tryptic soy agar (TSA), and incubated at 37°C overnight. A single colony was used to inoculate 25 ml of tryptic soy broth medium supplemented with 1% d-(+)-glucose (TSBG) and cultured overnight in preparation for biofilm experiments as described below.

### Growth and temperature conditions.

**FIG 6:**
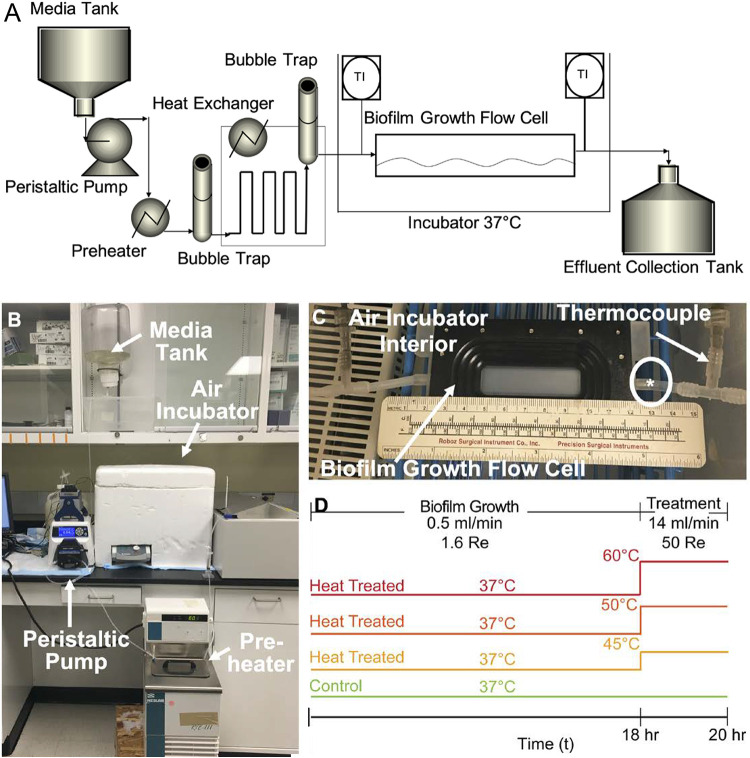
Flow cell system for temperature treatment of surface-adherent biofilms. (A to C) Schematic (A) and images (B and C) of the flow cell system used for temperature treatment of surface-adherent biofilms by fluid flow. Using a peristaltic pump, 10% TSBG is perfused through a series of heat exchangers and bubble traps, with the latter employed to remove dissolved gases. It then flows through a 4.7-mm (height) by 12.7-mm (width) by 49.3-mm (length) parallel plate flow chamber where the biofilm is grown, as shown in a close-up image in panel C. The fluid temperature is controlled by a heat exchanger, and the exterior is maintained at 37°C within an incubator at all times, including during temperature treatment. In panel C, the circled asterisk (*) indicates the inlet of the flow cell and media flow from right to left. (D) The experimental design. Biofilms were grown for 18 h at a flow rate of 0.5 ml/min. Following the growth period, the flow rate was increased to 14 ml/min with an accompanying temperature increase to 45°C, 50°C, or 60°C. In addition, a control experiment was performed at 37°C with increased flow only.

Panels A and B of Fig. 6 depict the biofilm culture system. Briefly, the system is comprised of silicon tubing through which medium is perfused though a series of heat exchangers and bubble traps as shown in Fig. 6A and B. The flow is driven by a peristaltic pump (Cole-Parmer, Vernon Hills, IL). Figure 6C shows the flow channel. Its dimensions are 2.35 mm (depth) by 12.7 mm (width) by 49.3 mm (length) (FC-81-PC flow cell; BioSurface Technologies Corp., Bozeman, MT). The flow cell chamber is polycarbonate, and a no. 1.5 coverslip is used for the imaging plane. The device was operated in the following way. One ml of overnight culture was diluted to an optical density of 0.25 ± 0.1 and injected into the point indicated by the circled asterisk (*) as shown in Fig. 6C. The flow cell was incubated at 37°C in air for 1 h without flow to allow adhesion. Sterile TSBG diluted 1:10 with sterile deionized (DI) water at 37°C was perfused through the flow cell at 0.5 ml/min for 18 h. This flow rate corresponds to a Reynolds number (Re) of 1.6 and a wall shear stress value of 0.00048 Pa. Here, the Reynolds number is calculated as follows:

(2) $$\text{Re}=\frac{\text{ρ}D_{h}v}{\text{μ}}$$

where ρ, *D_h_*, *v*, and μ are medium density, hydraulic diameter of the flow chamber, mean velocity of the media, and viscosity of the media, respectively. These conditions represent a common, physiologic, nonturbulent flow regime within the human body (31, 32). The duration of 18 h was selected to ensure mature biofilm coverage of the surface prior to treatment (28).

The treatment conditions, detailed below, were chosen to represent flow rates characteristic of the circulatory system. Figure 6D outlines the experimental design. After the initial growth phase, the flow rate of the media was increased to 14 ml/min (Re = 44). The wall shear stress of this flow was 0.01 Pa, near the lower range of the nonturbulent flow regime of moderately sized veins in the human body (33). This flow rate was maintained for 2 h. During these 2 h, the effects of heat stress on the biofilm were tested by changing the temperature of the media flowing inside the chamber to the conditions of 37°C, 45°C, 50°C, and 60°C (30). During this period, the outer surface of the flow cell continued to be incubated in air at 37°C. Heat transfer calculations (cf. Fig. SI.1, Equations SI.1 to SI.3, and Table SI.1) suggest that the temperature variability in the direction normal to the flow was no greater than 1.5°C across all experiments. For vancomycin experiments, the antibiotic was added to the media immediately prior to the 2-h time point at a concentration of 30 mg/liter. This concentration was chosen in order to mimic a therapeutic regimen (34, 35). The vancomycin MIC, defined as the lowest concentration of antibiotic necessary to inhibit visible bacterial growth following overnight culture, is typically ∼2 mg/liter for S. epidermidis (36).

The temperature at both the inlet and outlet of the flow cell was measured using type T thermocouples (Omega Engineering, Inc., Stamford, Connecticut). These temperature conditions were maintained such that the inlet and outlet temperatures were always within ±2°C of the target temperature. The flow rate of the experiments served to maintain nearly isothermal conditions across the length of the flow cell under all temperature conditions, as measured by the inlet and outlet thermocouples. The residence times were 3 s under growth conditions and 0.11 s during treatment. It took approximately 5 min to reach the steady-state temperature under treatment conditions.

### Confocal laser scanning microscopy (CLSM).

Following temperature treatment, biofilms were rinsed with deionized water and stained using a LIVE/DEAD BacLight bacterial viability kit (Molecular Probes Inc., Eugene, OR). The dye solution was prepared with a dye ratio of SYTO 9 to propidium iodide to deionized water of 3 μl:3 μl:2 ml, per manufacturer instructions. The dye mixture had a ratio of SYTO 9 to propidium iodide of 1:6 by concentration. After staining, the biofilms were incubated at room temperature for 60 min in the dark.

Stained biofilms were imaged using a Nikon A1RSi confocal laser scanning microscope, equipped with a CFI Plan Apo Lambda 100× oil lens objective with a numerical aperture (NA) = 1.45. The excitation wavelengths of the live and dead bacterial cell dyes are 488 nm and 561 nm, respectively. Fluorescein isothiocyanate (FITC) and Texas Red filters captured the emission spectra over 490 to 525 nm and 570 to 620 nm for live and dead cells, respectively. The image area was 30 by 30 μm^2^ in the objective plane, which was orientated parallel to the shearing surface. Image volumes were collected through a series of images up to a height of 10 μm perpendicular to the shearing surface. The voxel size was 0.062 by 0.062 by 0.062 μm. To ensure representative sampling of the flow cell biofilm, five image volumes were captured at points in the specimen corresponding to the shape of a cross. This cross was centered 20 mm from the flow cell entrance and 5 mm from the side wall of the flow cell. This location was selected to ensure that nutrient depletion had not occurred at the image point and that image collection was performed at locations far removed from those corresponding to potential effects of flow instabilities that might occur at the entrance, exit, edges, or corners of the flow cell. Image analysis was performed, in both two-dimensional (2D) and 3D modes, using trackpy (37), an image processing package implemented in python and based on the Crocker-Grier algorithm (38), to calculate the ratio of live to dead cells. The output of the image analysis was the centroid of each cell in the image or image volume.

For analysis of large-scale biofilm structures, 40-frame videos were taken while scanning a 1.0-cm-long section of biofilm. Images were obtained using a CFI Plan Apo Lambda 20× lens objective with NA = 0.75. Each frame was 512 × 512 pixels (px) (0.62 px/μm) and acquired 250 μm apart from its neighbors. Images were converted to the frequency domain using the fast Fourier transform (FFT) tool in ImageJ (39). The FFT intensity of each radial position was generated by averaging the intensity of fixed radius rings at up to 200 px in the frequency domain.

### Scanning electron microscopy.

Following confocal microscopy, the coverslip containing the biofilm sample was removed from the flow cell and submerged in a 2.5% glutaraldehyde solution. After a minimum of 24 h, the sample was washed and dried using serial washes with increasing concentrations of ethanol. Samples were mounted on an SEM stub, sputter coated with gold, and imaged (FEI Nova 200 nanolab SEM/FIB).

### Quantitative growth culture.

Following the growth and temperature treatment phases detailed above, biofilm samples were removed from the glass surface by a combination of scraping and washing with 25 ml DI water. The glass slide was then sonicated for 5 min to ensure the removal of all adhered cells (40). The resulting liquid/biofilm mixture was homogenized (Ika Ultra-Turrax T18 basic homogenizer) at 18,000 rpm for 1 min. The total number of cells was then quantified by 10-fold serial dilution and plating on agar. The minimum limit of detection for this assay was found to be 24 CFU per mm^2^.

### Growth curve of downstream media containing sloughed and debrided cellular material.

All of the media used for the 2-h temperature treatment was sterilely collected as effluent. This was accomplished by connecting a sterile carboy to the flow cell waste stream at the beginning of the treatment as shown in Fig. 6A (marked “Effluent Collection Tank”). A 1.68-liter volume of previously heated effluent was collected and stored at room temperature. Following collection, the effluent was vigorously mixed to ensure an even distribution of cells throughout the solution. Two measurements were collected, (i) 2 ml of effluent and (ii) 1 ml of effluent added to 1 ml of fresh TSBG in the channel of a 12-well plate, and the optical density at 600 nm (OD_600_) was recorded for 18 h using a multimode microplate reader (Synergy 2; BioTek). The two measurements served different purposes. The undiluted effluent was used to assess the initial OD. The diluted effluent included new growth medium, to accommodate the possibility that the original 10% medium was spent. Adding fresh medium introduced fresh nutrients in a way that represented a better opportunity to assess the viability of the disseminated cells.

## Supplementary Material

Supplemental file 1
