# Supplementary material for: Differential Effects of Heated Perfusate on Morphology, Viability, and Dissemination of Staphylococcus epidermidis Biofilms
Source: Appl Environ Microbiol. 2020 Oct 1;86(20):e01193-20. doi: 10.1128/AEM.01193-20 (PMC7531952; doi:10.1128/AEM.01193-20)
Supplement: Supplemental file 1 [file AEM.01193-20-s0001.pdf]

SUPPLEMENTAL: Differential effects of heated perfusate on morphology, viability, and dissemination of *Staphylococcus epidermidis* biofilms

Joanne Beckwith, J. Scott VanEpps, Michael J. Solomon

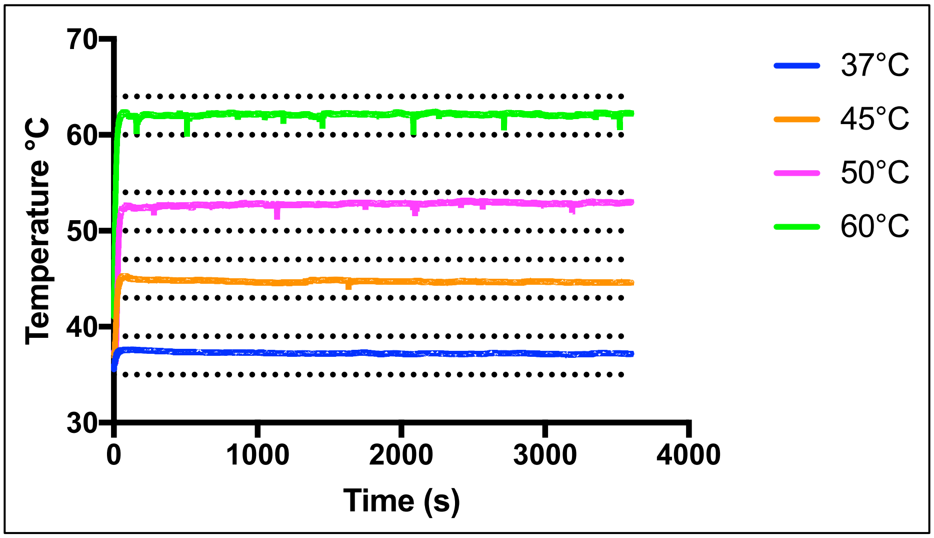

Figure SI.1: Temperature profiles for the heated fluid exposed to biofilms. Solid lines represent the average of the inlet and outlet thermocouples. After reaching steady state, all temperature readings were within the ±2° range noted by the dashed lines. All treatments reached a steady state after approximately 5 minutes.

To further support that the set point temperatures are indicative of the temperature of the perfusate and biofilm in the flow cell, a heat transfer calculation was performed. The calculation modeled the heat transfer coefficient at the inner surface of the flow cell as for laminar flow of water at the treatment temperature and at the outer surface as for stagnant air at 37°C with natural convection. Although the calculation does include the biofilm physical properties, it does provide a useful bound on the temperature profile. Energy balances for the overall system, the inner surface, and the outer surface are reported in equation SI.1, SI.2, and SI.3, respectively (McCabe, Smith, Harriott, Unit Operations for Chemical Engineering, 7th edition, 2005.) Table SI.1 reports the inner and outer wall temperatures, Tw1 and Tw2, respectively. The inner wall temperature never deviates from the set point temperature by more than 1.5°C.

$$\frac{Q}{A} = h_1(T_{w1} - T_1) = h_2(T_2 - T_{w2}) = \frac{k}{L}(T_{w2} - T_{w1}) \qquad \text{Eqn. SI.1}$$

$$Nu = 0.664\sqrt[3]{Pr}\sqrt{Re} \qquad \text{Eqn. SI.2}$$

$$\frac{hL}{k} = b\left[\frac{L^3\rho^2 g\beta\Delta T}{\mu^2}\left(\frac{C_p\mu}{k}\right)\right]^n \qquad \text{Eqn.SI.3}$$

Q Quantity of heat [J]
A Area of heat transfer [m^2]
h Individual surface heat-transfer coefficient [W/m^2]; for air side surface (flow cell outer surface) h_1; for water side surface (flow cell inner surface) h_2.
T Bulk Temperature [°C]; for air side surface (flow cell outer surface) T_1; for water side surface (flow cell inner surface) T_2.
T_w Wall temperature [°C]; for air side surface (flow cell outer surface) T_{w1}; for water side surface (flow cell inner surface) T_{w2}.
k Thermal conductivity [W/m]
L Length [m]
Nu Nusselt number [Dimensionless]
Pr Prandtl number [Dimensionless]
Re Reynolds number [Dimensionless]
b Constant- Natural convection for horizontal plate*
ρ Density [kg/m^3]
g Acceleration due to gravity [m/s^2]
β Coefficient of thermal expansion of fluid [1/K]
μ Absolute viscosity [mPa*s]
C_p Specific heat at constant pressure [J/°C]
n Constant exponent value for natural convection for horizontal plate*

*From W.L. McCabe, J.C. Smith, P. Harriott, Unit Operations of Chemical Engineering 7th edition., p 380. McGraw-Hill 2005.

	T_{w1} (°C)	T_{w2} (°C)
37°	37	37
45°	44.9	44.6
50°	49.9	49.2
60°	59.9	58.4

Table SI.1 contains the final results for the wall temperatures at each of the treatment conditions.

	a_1	b_1 (μm)	a_2	b_2 (μm)	d	a_1/a_2
37°	32.3 ± 4.8	5.7 ± 1.4	70.7 ± 18.8	0.22 ± 0.06	75.7 ± 17.6	0.46 ± .15
45°	23.3 ± 3.2	7.0 ± 1.6	53.4 ± 8.5	0.5 ± 0.05	102.6 ± 5.7	0.44 ± 0.14
50°	37.1 ± 6.9	6.1 ± 1.4	57.9 ± 10.9	0.27 ± 0.02	93.4 ± 7.0	0.64 ± 0.28
60°	36.1 ± 5.3	7.4 ± 1.8	45.1 ± 10.0	0.61 ± 0.35	103.1 ± 9.7	0.80 ± 0.33

Table SI.2 Initial FFT five parameter model fit parameters

	a_1	b_1 (μm)	a_2	b_2 (μm)	d	a_1/a_2
37°	28.5 ± 6.9	7.59	51.4 ± 2.4	0.3475	99.1 ± 4.0	0.56 ± 0.14
45°	25.4 ± 4.2	7.59	59.5 ± 9.2	0.3475	94.4 ± 5.7	0.48 ± 0.16
50°	33.2 ± 5.8	7.59	59.4 ± 9.9	0.3475	95.7 ± 7.3	0.63 ± 0.23
60°	40.0 ± 4.8	7.59	38.3 ± 2.4	0.3475	106.0 ± 1.9	1.1 ± 0.18

Table SI.3 b_1 and b_2 fixed value FFT model fit parameters

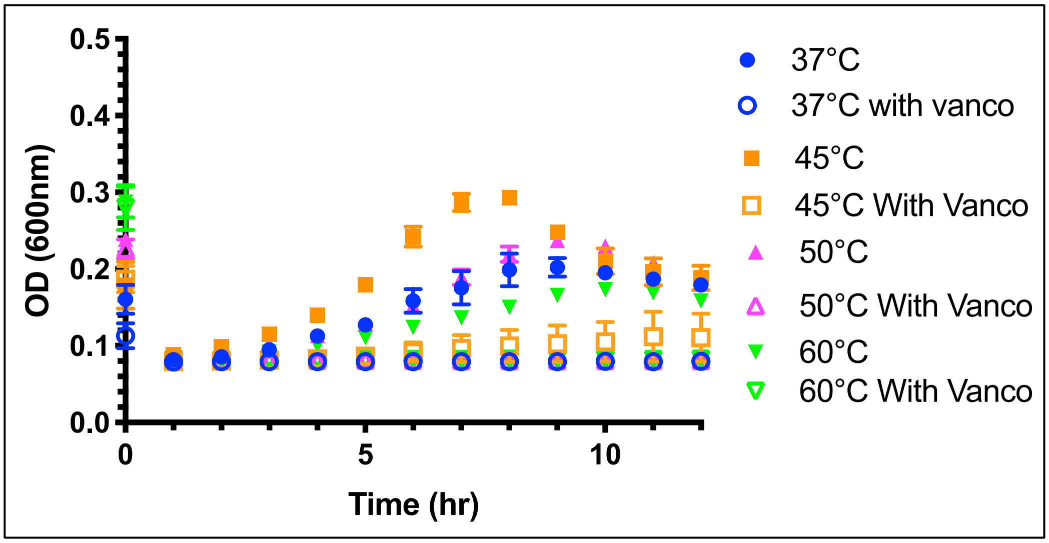

Figure SI.2: Growth curve for 2 mL effluent with the additional presence of vancomycin.
